# Activity and Crystal Structure of the Adherent-Invasive *Escherichia coli* Tle3/Tli3 T6SS Effector/Immunity Complex Determined Using an AlphaFold2 Predicted Model

**DOI:** 10.3390/ijms24021740

**Published:** 2023-01-16

**Authors:** Thi Thu Hang Le, Christine Kellenberger, Marie Boyer, Pierre Santucci, Nicolas Flaugnatti, Eric Cascales, Alain Roussel, Stéphane Canaan, Laure Journet, Christian Cambillau

**Affiliations:** 1 Architecture et Fonction des Macromolécules Biologiques, Aix Marseille University and CNRS, 13288 Marseille, France; 2 Laboratoire d’Ingénierie des Systèmes Macromoléculaires (LISM), Institut de Microbiologie, Bioénergies et Biotechnologie, Aix Marseille University and CNRS, 13009 Marseille, France; 3 AlphaGraphix, 66210 Formiguères, France; 4 School of Microbiology, University College Cork, T12 K8AF Cork, Ireland

**Keywords:** type VI secretion system, phospholipase, immunity, adherent-invasive *Escherichia coli* (AIEC), protein secretion, X-ray structure, AlphaFold2

## Abstract

The type VI secretion system (T6SS) delivers enzymatic effectors into target cells to destroy them. Cells of the same strain protect themselves against effectors with immunity proteins that specifically inhibit effectors. Here, we report the identification and characterization of a Tle3 phospholipase effector and its cognate immunity protein Tli3—an outer membrane lipoprotein from adherent-invasive *Escherichia coli* (AIEC). Enzymatic assays demonstrate that purified Tle3^AIEC^ has a phospholipase A1, and not A2, activity and that its toxicity is neutralized by the cognate immunity protein Tli3^AIEC^. Tli3^AIEC^ binds Tle3 in a 1:1 stoichiometric ratio. Tle3^AIEC^, Tli3^AIEC^ and the Tle3^AIEC^-Tli3^AIEC^ complex were purified and subjected to crystallization. The Tle3^AIEC^-Tli3^AIEC^ complex structure could not be solved by SeMet phasing, but only by molecular replacement when using an AlphaFold2 prediction model. Tle3^AIEC^ exhibits an α/β-hydrolase fold decorated by two protruding segments, including a N-terminus loop. Tli3^AIEC^ displays a new fold of three stacked β-sheets and a protruding loop that inserts in Tle3^AIEC^catalytic crevice. We showed, experimentally, that Tle3^AIEC^ interacts with the VgrG ^AIEC^ cargo protein and AlphaFold2 prediction of the VgrG^AIEC^-Tle3^AIEC^ complex reveals a strong interaction between the VgrG^AIEC^ C-terminus adaptor and Tle3^AIEC^ N-terminal loop.

## 1. Introduction

The type VI secretion system (T6SS) is a macromolecular machine anchored in the envelope of Gram-negative bacteria. This nanomachine allows the injection of effectors or toxins into eukaryotic or bacterial target cells and is involved in virulence and antibacterial competition. T6SS^+^ cells that deliver antibacterial effectors also produce specific immunity proteins to protect themselves from the toxicity of these effectors.

The subunits required to assemble a functional T6SS machine are usually encoded within gene clusters, together with additional toxin and immunity genes. These subunits assemble a membrane complex embedded in the envelope that anchors a cytoplasmic contractile tail structure [1,2,3,4]. The contractile tail is an internal tube made of Hcp protein hexamers, surrounded by a sheath-like structure and tipped by a needle-spike. The spike is built by a trimeric protein named VgrG, topped by a conical protein named PAAR [5,6]. The contractile tail assembles on a baseplate platform in an elongated conformation [7,8,9]. Upon contact with the target bacterium, the contraction of the sheath outwardly propels the tube and spike directly into the target cell. Because the toxins can be fused to or interact with the Hcp tube component or the VgrG/PAAR spike proteins, the contraction of the sheath leads to the secretion and delivery of the effectors into the target cell.

Antibacterial toxins’ injection into the cytoplasm or the periplasm of target bacteria leads to bacterial cell lysis or growth inhibition. Cytoplasmic-acting antibacterial effectors include DNases, deaminases, NAD(P) + glycohydrolase toxins, p(p)pApp synthetase and post-translational modification enzymes, such as ADP-ribosyltransferases [10,11]. Periplasmic-acting antibacterial effectors include enzymes degrading the peptidoglycan, such as amidases or glycosides hydrolases, or toxins targeting the membrane, such as pore-forming toxins or phospholipases [10,11].

Type VI lipase effectors (Tle) were classified in five families (Tle1-5) [12]. Members of the Tle5 family, such as *Pseudomonas aeruginosa* PldA and PldB, are phospholipases D [12,13,14]. Members of families 1 to 4 (Tle1–4) bear a GxSxG motif and a putative catalytic triad (S-H-D). *B. thailandensis* Tle1^BT^ and *P. aeruginosa* Tle1^PA^ demonstrate phospholipase A2 (PLA2) activity [12,15], while *Vibrio cholerae* Tle2^VC^ (TseL) and *P. aeruginosa* Tle4^PA^ (TplE) demonstrate phospholipase A1 (PLA1) activity [12,16]. Tle1^EAEC^ was shown to possess PLA1 activity and, to a lesser extent, PLA2 activity [17]. The enzymatic activity of Tle3 family members has not been determined as yet. All Tle characterized effectors are antibacterial effectors, likely degrading the membrane phospholipids of target cells. Interestingly, T6SS phospholipases can demonstrate activity in eukaryotic cells, and thus are considered as transkingdom effectors [13,16].

We previously characterized Tle1 and Tli1, a toxin/immunity couple of enteroaggregative *Escherichia coli* (EAEC) [17]. Tle1 is a periplasmic-acting toxin with phospholipase A1/A2 activity that mediates the antibacterial activity of EAEC T6SS-1. Tle1^EAEC^ activity is counteracted by Tli1^EAEC^, a small outer membrane lipoprotein encoded downstream from the *tle1* gene. We further showed that the delivery of Tle1^EAEC^ into bacteria requires a direct interaction of Tle1^EAEC^ with the VgrG1 protein [17]. The EAEC VgrG1 is comprised of a gp27-like base, gp5 β-helix and a C-terminal extension with a transthyretin (TTR) fold. We recently determined the high-resolution structure of the VgrG1/Tle1 complex by cryo-EM, demonstrating that Tle1^EAEC^ binds to the VgrG1 TTR domain and that the interaction is stabilized by additional contacts with the VgrG β-helix and base [18].

The presence of phospholipase/immunity genes is a conserved feature of T6SS-1 clusters in *E. coli* strains [19]. However, these phospholipase effectors belong to different families (Tle1 for EAEC, Tle4 for avian pathogenic *E. coli* and a predicted Tle3 for uropathogenic *E. coli* CFT073 or adherent-invasive *E. coli* (AIEC) [12,17,19,20]). AIEC is implicated as a determinant of human Crohn’s disease (CD) development, as ileal gut mucosa colonization by AIEC is often observed in CD patients [21,22].

Here, we biochemically and structurally characterized Tle3 from AIEC and its cognate Tli3 immunity protein. We showed that Tle3^AIEC^ has PLA1 activity that is specifically counteracted by the Tli3^AIEC^ immunity protein. We solved the structure of the Tle3^AIEC^/Tli3^AIEC^ complex by X-ray crystallography, with the help of Alphafold2 structure predictions [23,24]. Finally, a model of a Tle3^AIEC^/VgrG ^AIEC^ complex was proposed.

## 2. Results

### 2.1. Genomic Context and Bioinformatic Analyses of AIEC T6SS-1 Phospholipase Effector/Immunity

**Figure 1:**
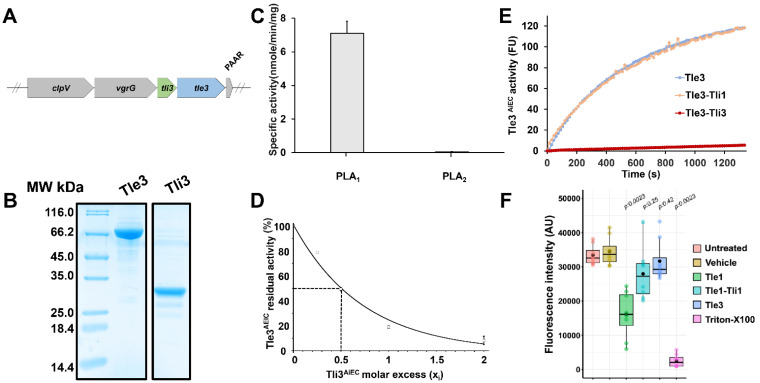
Biochemical characterization of recombinant Tle3^AIEC^. (**A**) Schematic representation of the genetic organization of vgrG, tli3 and tle3 genes from AIEC LF82 T6SS-1 cluster. The LF82_434 and LF82_435 genes encoding Tli3^AIEC^ and Tle3^AIEC^ are indicated in green and blue, respectively. The clpV, vgrG and PAAR T6SS-1 genes are indicated in grey. (**B**) The SDS-PAGE analysis of the purified Tle3^AIEC^ and Tli3^AIEC^ proteins (10 µg) after Coomassie blue staining. Molecular weight markers from Euromedex (in kDa) are indicated on the left. (**C**) Specific phospholipase A1 (PLA1) and A2 (PLA2) activity measurements of the Tle3^AIEC^ protein using fluorescent phospholipids. Specific activities were calculated from the velocity slope obtained for 25 min using 20 µg of purified protein. Data are expressed as mean of three, from three independent assays (CV% < 5%). (**D**) Inhibition studies with Tli3^AIEC^ were performed by incubating 30 µg of Tle3^AIEC^ with various molar ratio of Tli3^AIEC^ (x_i_ = 0, 0.25, 0.5, 1 and 2). The rate of hydrolysis of PED-A1 by purified Tle3^AIEC^ at 20 °C in presence of increasing concentrations of Tli3^AIEC^ was plotted against the molar excess of Tli3. (**E**) Cross-inhibition studies with Tli1^EAEC^ were performed by incubating 20 µg of Tle3^AIEC^ (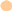) with a molar ratio of 10, 20 µg of Tle3^AIEC^ alone (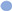) or incubated with Tli3^AIEC^ (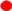) at molar ratio of 2, were used as controls. (**F**) Cytotoxic activities of Tle1^EAEC^ and Tle3^AIEC^ towards eukaryotic cells. Cytotoxicity experiments were carried with approximately 1 × 10^5^ RAW264.7 cells/well pulsed with Tle1^EAEC^ and Tle3^AIEC^ recombinant proteins (30 µg) incubated for 16 h. Tle1^EAEC^ phospholipase A2 and lysis buffer containing 1% Triton X-100 were used as positive controls. Alternatively, cells were incubated with medium containing the vehicle buffer or the Tle1^EAEC^-Tli1^EAEC^ complex as additional negative controls. Viability was evaluated using resazurin assay. Results are displayed as box-plots with individuals’ raw data. Black dots were added to highlight the mean of each condition. Results are representative from n = 2 biologically independent experiments performed in technical quadruplicates. Statistical analysis was performed using Wilcoxon signed-rank test, where vehicle treatment was used as reference condition. Exact adjusted *p*-value are indicated on the graph and were considered significant when *p* ≤ 0.05.

*E. coli* T6SS-1 clusters encode similar machines, but different effector/immunity pairs are encoded in between the vgrG and PAAR genes. The AIEC reference strain LF82 encodes a putative Tli3/Tle3 immunity/toxin couple (Figure 1A). The tle3^AIEC^ gene (LF82_435, NCBI Gene Identifier (GI): 222034514) is predicted to encode a 658-aminoacid (73.9 kDa) cytoplasmic protein identical to the uncharacterized c3395 protein from *E. coli* CFT073, classified by Russell et al. as a member of the Tle3 family of T6SS effectors [12]. Alignment of Tle3 members reveals the presence of a conserved XHSQG motif (G/S/C/A-H-S-Q-G motif in Tle3) (Figure S1).

In T6SS, protection against kin cells that produce toxic effectors is ensured by the production of immunity proteins that specifically bind and inhibit their corresponding toxin. Effector/immunity genes are found in tandem in genomes, and immunity proteins of Tle effectors, named Tli (Type VI lipase immunity), are usually periplasmic proteins or lipoproteins, as Tle are periplasmic-acting toxins. Computer analysis of the LF82_434 gene product (GI: 222034513) using SignalP 6.0 suggests that LF82_434 is a lipoprotein with a signal sequence bearing a characteristic lipobox motif with an isoleucine as +2 residue, suggesting an outer membrane localization (Figure S2; [25]). A fractionation experiment confirmed that LF82_434 co-localizes with the membrane fraction (Figure S2). Its localization and genomic context close to tle3 suggest it could correspond to the Tle3 immunity protein; therefore, it was named Tli3.

### 2.2. Tle3^AIEC^ and Activity and Inhibition by Tli3^AIEC^

T6SS Tle1, Tle2 and Tle4 effectors were demonstrated to have phospholipase A1 (PLA1) or phospholipase A2 (PLA2) activity [12,15,16]. Interestingly, Tle1^EAEC^ has both phospholipase A1 and phospholipase A2 (PLA2) activities [17]. By contrast, no enzymatic activity has been assigned, to date, for any member of the Tle3 family.

The AIEC Tle3 protein, Tle3^AIEC^, was purified (Figure 1B), and its activity was tested using fluorogenic PLA1- and PLA2-specific phospholipid substrates. As shown in Figure 1C, Tle3^AIEC^ possesses phospholipase A1 (PLA1) activity (specific activity (SA) = 7.65 nmole.min^−1^.mg^−1^) but no PLA2 activity. This result supports our previous remark that Tle classification was based on their protein sequences and phylogenetic distribution, and not on their activity [17].

The Tli3^AIEC^ putative immunity, devoid of is signal sequence and N-terminal cysteine residue, was purified (Figure 1B), and tested for its ability to interfere with Tle3^AIEC^ activity. Thirty micrograms of Tle3^AIEC^ were incubated with various molar ratios of Tli3^AIEC^ (xi = 0, 0.25, 0.5, 1 and 2), and the activity was measured immediately or after 30 min incubation at room temperature. Figure 1D shows that the Tle3^AIEC^ phospholipase activity was inhibited by Tli3^AIEC^ in a dose-dependent manner. Interestingly, the Tle3^AIEC^ activity was nearly abolished with a Tle3^AIEC^:Tli3^AIEC^ molecular ratio of 1:2 (Figure 1D). Tli3^AIEC^ is thus likely to interact strongly with Tle3^AIEC^, probably inside or in a close vicinity to the active site, since no substrate can have access to the active site to be hydrolyzed. These results indicate that Tli3^AIEC^ is an immunity protein that inhibits the phospholipase activity of Tle3^AIEC^.

To better understand the specificity of Tli3 towards Tle3, cross-inhibition assays with Tli1^EAEC^ (from EAEC T6SS-1, [17]) were performed by incubating 20 µg of Tle3^AIEC^ with various molar ratios of Tli1 (x_i_ = 0, 0.5, 1, 2 and 10) (Figure 1E). No inhibition by Tli1^EAEC^ was detected even with the highest immunity/effector ratio. As expected, this result indicates specificity between Tle and Tli, without any cross-reaction.

Because AIEC is a human pathogen, we then investigated whether Tle3^AIEC^ is cytotoxic in mammalian cells (Figure 1F). Many bacterial enzymes presenting phospholipase A2/A1 activity possess cytotoxic activity against mammalian cells [26,27]. In order to estimate the effect of Tle3^AIEC^ on a RAW 264.7 cell, we used the well-established resazurin-based cell viability assay. As controls, lysis buffer containing Triton X-100 leads to 100% cellular lysis while the untreated and buffer-only conditions maintained cell viability. The addition of the Tle3^AIEC^ PLA1 did not show any significant cytotoxicity (~15%) towards mouse macrophages. In contrast, the incubation with the Tle1^EAEC^ PLA1/PLA2 led to ~50% lysis of the RAW 264.7 cells in 16 h. This cytotoxic effect can be inhibited by preincubating Tle1^EAEC^ with Tli1^EAEC^, its cognate and specific immunity. Our results are in agreement with the literature, supporting the idea that the PLA2 activity is required for a cytotoxic effect towards mammalian cells [28]. Finally, these results confirm the biochemical properties of Tle3^AIEC^ as a PLA1 enzyme.

### 2.3. Tle3^AIEC^-Tli3^AIEC^ Heterodimer Crystallization and Data Collection

The purified Tle3^AIEC^ and Tli3^AIEC^ proteins were mixed with a molar ratio of 1:1.3. The complex was then collected by gel filtration chromatography (Supplementary Figure S3). The Tle3^AIEC^-Tli3^AIEC^ complex has an apparent molecular mass of 100 kDa (Supplementary Figure S3C). Considering its theoretical molecular weight of 101.9 kDa, it is likely to be a 1:1 Tle3^AIEC^-Tli3^AIEC^ heterodimer in solution.

Highly purified protein fractions were pooled and concentrated to 5.6 mg/mL. The Tle3^AIEC^-Tli3^AIEC^ SeMet purified complex was then submitted to crystallization. Sharp crystals appeared within 10 days to 3 weeks, reaching maximum dimensions of 100 × 40 × 40 µm within 2 months. The best crystals were exposed at the Soleil synchrotron (Saint-Aubin, France) and diffracted to 3.6-Å resolution (Table 1). Tle3^AIEC^-Tli3^AIEC^ crystals belong to space group P21, with unit-cell parameters a = 67.9 Å, b = 449.1 Å, c = 116.2 Å and β = 91.2° (Table 1). The crystals exhibit a Vm of 17.4 Å^3^/Da for 1 complex per asymmetric unit. Based on the molecular weight of 101,9 kDa, a total of 4 to 8 complexes per asymmetric unit should be expected, as this would lead to more reasonable values of Vm, in the range of 4.35–2.17 Å^3^/Da, and solvent contents of 72–43%. However, due to the low redundancy of the collected data set, data resolution, large cell and 4-8 complexes of ~900 amino acids in the asymmetric unit, the structure could not be solved by SeMet phasing nor with molecular replacement, and the project was abandoned for 6 years.

### 2.4. Crystal Structure Determination Using Molecular Replacement with AlphaFold2 Models

Recently (15 July 2021) AlphaFold2 (AF2) [23], a highly efficient protein structure prediction program, was released. With the idea to resuscitate the project, we submitted the sequences of Tle3^AIEC^ and Tli3^AIEC^ to AlphaFold2 and obtained models with satisfying statistics (pLDDT > 80; Supplementary Figure S4). However, since some loops or stretches of residues were poorly predicted, we removed them from the starting models used for molecular replacement (Supplementary Figure S5). Molecular replacement with Molrep [29] using the AF2 “cleaned” model yielded four Tle3^AIEC^ molecules in a first pass. This starting model was refined with Phenix [30] and a second pass of molecular replacement yielded three more molecules. After refinement, the seven-molecule ensemble was used as the fixed model for molecular replacement with Tli3^AIEC^, yielding two Tli3^AIEC^ molecules. The Tle3^AIEC^-Tli3^AIEC^ complex was substituted on the six remaining Tle3^AIEC^, and the seven complexes were subjected to refinement with Phenix. After cycles of model building with Coot [31] and Phenix refinement [30], an eight-complex ensemble was identified and a new cycle of refinement was performed. Finally, the 8 complexes exhibited a reasonable geometry for a 3.6 Å resolution with R and R_free_ factors of 23.4% and 26.8%, respectively (Table 1).

### 2.5. Structure of Tle3^AIEC^ and Tli3^AIEC^

**Figure 2:**
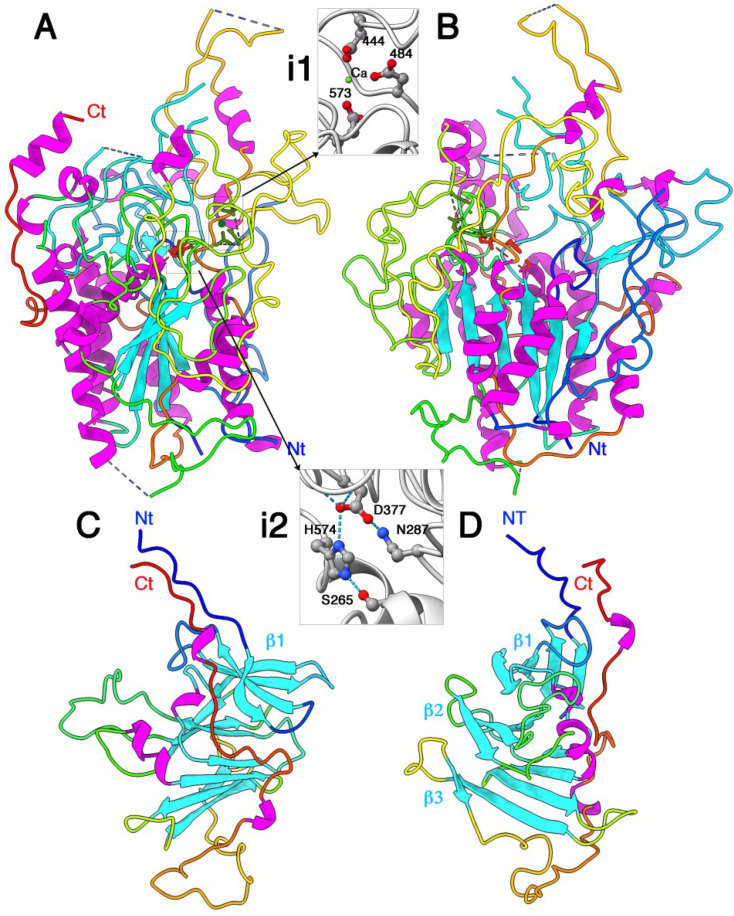
X-ray structure of Tle3^AIEC^ and Tli3^AIEC^. (**A**) Structure of Tle3^AIEC^; the catalytic triad is colored red and the Ca^2+^ cation in green. (**i1**) The Ca^2+^ ligands. (**i2**) The catalytic triad. (**B**) Same view as (**A**) rotated 90° around a vertical axis. (**C**) Structure of Tli3. (**D**) Same view as (**D**) rotated 90° around a vertical axis. The three β-sheets are labeled β1–3. (**A**–**D**) The protein backbones are rainbow colored. The helices are colored magenta and the β-strands are colored blue. The inset atoms arec colored red (oxygen), blue (nitrogen), grey (carbon) and green (calcium).

The Tle3^AIEC^ phospholipase chain is 658 residues long, of which 525, on average, have been identified in the electron density map. Tle3^AIEC^ structure is bulky, with overall dimensions of ~75 Å × 65 Å × 55 Å (Figure 2A,B). Stretches in the amino acid chain are lacking at the N- and C-termini, as well as in some more or less extended loops. Tle3^AIEC^ shares the classical core of an α/β-hydrolase fold [32], with a β-sheet of six parallel β-strands in the order β_2_β_1_β_3_β_4_β_5_β_6_. The β-sheet is covered by two α-helices on one face, and five helices on the other face. Very large decorations, in which structured elements are a minority, complete the structure (Figure 2A,B). A cation, probably Ca^2+^, is observed at bonding distance from three acidic side chains from Asp444, Asp484 and Asp573 and the C=O group of Asp377 (Figure 2, inset 1; Supplementary Figure S6a). In enzymes belonging to the α/β-hydrolase fold, the nucleophilic serine is located at the tip of a sharp β–strand/α–helix kink with a GxSxG consensus sequence. In Tle3^AIEC^, the catalytic serine was identified in a short loop between a β–strand and a α–helix, with the sequence SHSQG at position 265. Notably, the first glycine of the consensus kink sequence GxSxG is replaced here by a serine. As in all α/β-hydrolase fold, the Ser exhibits a special position in the Ramachandran graph, with Phi and Psi angles of 60° and −135°, respectively. The Ser265 hydroxyl moiety is implicated in a hydrogen bound with the Ne2 atom of His574 which is, in turn, hydrogen-bound by its Nd1 atom to the Asp377 carboxyl group, forming the catalytic triad (Figure 2, inset 2; Supplementary Figure S6b). An Asn side chain further orientates the carboxyl moiety of Asp377. Notably, Asp377 C=O group participates in the binding of the Ca^2+^ cation, whose function might be to stabilize the catalytic triad geometry. The oxyanion hole, a device that stabilizes the oxyanion transition state via hydrogen bonds with two main-chain nitrogens, is often evidenced by using organophosphate or organophosphonate complexes. Two main-chain NH groups establish short hydrogen bonds with an oxygen belonging to the phosphonate group. In Tle3^AIEC^, in the absence of bound inhibitors, the oxyanion hole has been identified on the basis of comparisons with other α/β-hydrolases. Within the α/β hydrolase fold family, one obligate component of the oxyanion hole is the NH group of the residue following the nucleophile Ser (Gln266 in Tle3^AIEC^). The second NH group occupies a different position in sequence but always originates from the same spatial region. Here, by comparison with gastric lipase in complex with a phosphonate inhibitor ([33], PDB id: 1k8q), we identified Val55 as the second NH group involved in the oxyanion hole.

The catalytic triad is located at the bottom of a crevice ~30-Å deep and ~20 × 15 Å (Figure 2A). Surprisingly, the walls of the crevice, as well as the surface residues around it, are not particularly hydrophobic. A Dali search with Tle3^AIEC^ structure returned hits with a palmitoyl thioesterase (PDB id 1pja; Z = 12.1; r.m.s.d. = 3.3 Å; aligned 187) and a lipase (PDB id 5h6g; Z = 11.4; r.m.s.d. = 3.1; aligned 176). Indeed, most residues belonging to the α/β-hydrolase fold core, i.e., the six β–strands and the seven α–helices, are aligned. Comparison with the apo- and phosphonate-complexed gastric lipase suggests that the oxyanion hole is preformed, even without substrate.

The Tli3^AIEC^ immunity protein is predicted to be translated as a 274-residue long protein, with 24 residues cleaved upon processing and acylated at the N-terminal cysteine residue of the mature form (Figure S2). A total of 30–33 residues at the N-terminus of the mature form, which may correspond to a linker, as well as 0–5 residues at the C-terminus, are not observed in the electron density map. The Tli3^AIEC^ structure possesses overall dimensions of 60 × 45 × 45 Å. (Figure 2C,D). The core of Tli3^AIEC^ is assembled in three stacked β-sheets of 4, 3 and 4 β-strands, respectively, with topology of β_1_β_2_β_3_β_4_, β_6_β_7_β_8_, β_5_β_10_β_11_β_12_. A long loop of 17 residues is observed between strands β6 and β7 (residues 133–150). A large loop after strand β_12_ is followed by a very long extended C-terminal segment that runs antiparallel to the extended N-terminus. The extremities of these segments point outwards from the core. Dali returned several hits with small Z values (~6–6.4) and a relatively low number of residues aligned (~120). These hits cover many different functions and reflect only resemblances between the β structures. Overall, the fold of Tli3^AIEC^ seems unique.

### 2.6. Structure of the Tle3^AIEC^-Tli3^AIEC^ Complex

**Figure 3:**
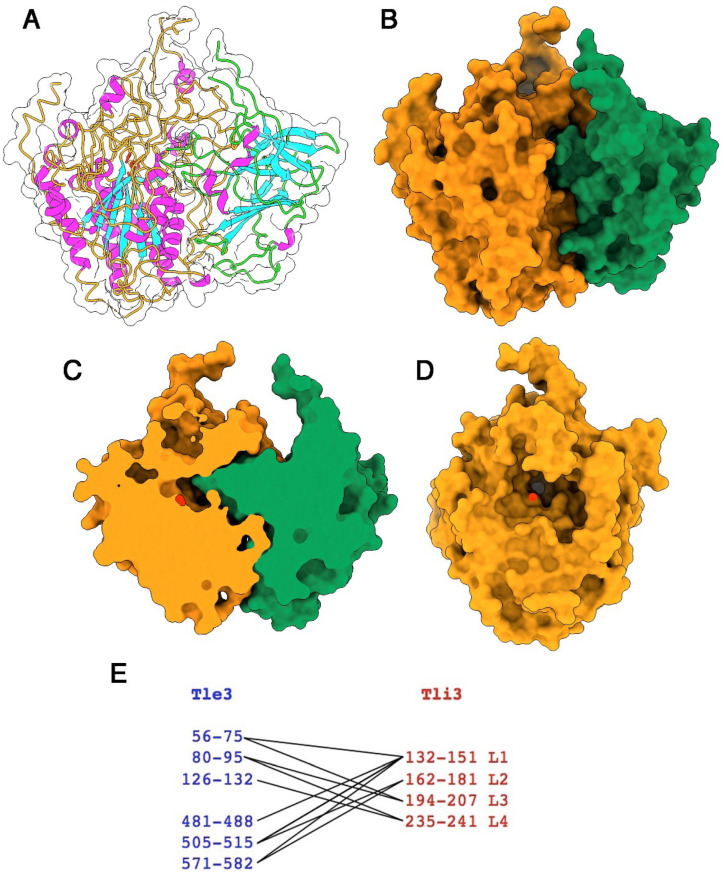
Structure of the Tle3^AIEC^/Tli3^AIEC^ complex. (**A**) Ribbon view and protein surface (white transparent) of Tle3^AIEC^ backbone colored orange and Tli3^AIEC^ backbone colored green. The catalytic triad is colored red, helices and strands are colored purple and blue, respectively. (**B**) Molecular surface of the Tle3^AIEC^/Tli3^AIEC^ complex (same colors as in (**A**). (**C**) Molecular surface of the Tle3^AIEC^/Tli3^AIEC^ complex cut at the catalytic triad (red) level. Same orientation as in (**B**). (**D**) Molecular surface of Tle3^AIEC^ rotated 90° as compared to (**B**) evidencing the catalytic crevice with the catalytic triad position indicated at its bottom (red). (**E**) Schematic representation of the Tle3^AIEC^ (blue) and Tli3^AIEC^ (red) interacting in the complex.

The Tle3^AIEC^-Tli3^AIEC^ complex has overall dimensions of ~85 Å × 75 Å × 55 Å. The β_6_-β_7_ Tli3^AIEC^ loop inserts deeply in the Tle3^AIEC^ substrate-binding crevice, without reaching catalytic Ser265, however (Figure 3A–D). A water surface area of 1800 Å^2^ is buried upon complex formation, as calculated by Pisa server [34], which represents 11% of the total Tle3^AIEC^ surface and 28% of the Tli3^AIEC^ surface. Four loops of Tli3^AIEC^ interact with the Tle3^AIEC^ crevice, residues 132–151 (Loop1), 162–181 (Loop 2), 194–207 (Loop 3) and 235–241 (Loop 4) (Figure 3E). Loops 1 and 2 interact mainly with the C-terminal part of Tle3^AIEC^ (residues 481–582), while loops 3 and 4 interact mainly with the N-terminal part (residues 56–132).

### 2.7. Comparison of the X-ray- and AlphaFold2-Predicted Structures

**Figure 4:**
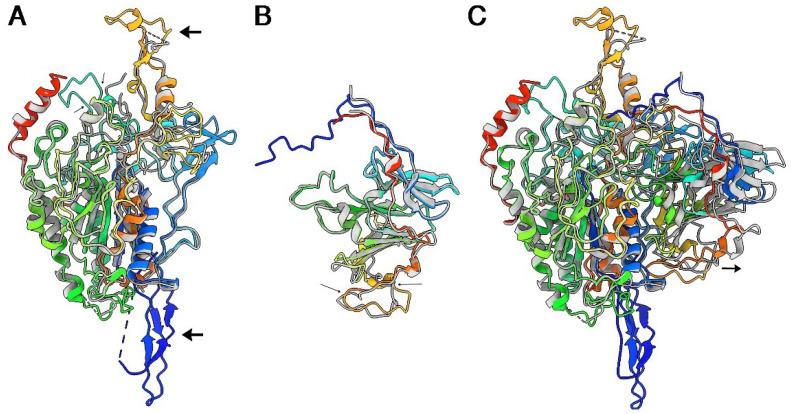
Comparison of X-ray- and AF2-predicted structures. Superposed ribbon views of (**A**) Tle3^AIEC^, (**B**) Tli3^AIEC^ and (**C**) Tle3^AIEC^/Tli3^AIEC^ complex. The small arrow indicates the positional difference between the X-ray structure and the predicted structure. (**A**–**C**) The X-ray structures are colored grey and the predicted structures are rainbow-colored.

Superposition of AF2 prediction of Tle3^AIEC^ onto the crystal structure yields an r.m.s.d of 1.12 Å (528/555 residues) (Figure 4A). Two predicted loops are absent in the crystal structure between residues 203–215 and residues 537–552. The AF2 prediction of Tle3^AIEC^ exhibits two protruding domains at opposite sides of Tle3^AIEC^ core that are not visible in the crystal structure, probably due to dynamic disorder. One of these extensions is the above-mentioned loop, 537–552, that starts and terminates with two antiparallel β-strands. The second is formed by the N-terminus of Tle3^AIEC^ (residues 1–43), including a poorly predicted stretch of ~10 residues followed by a well-predicted structure of 3 extended stretches interlocked by 4 β-strands (Figure 4A). The tip of these two extensions, as well as loop 203–215, display lower prediction statistics (Supplementary Figure S4), which together with their absence in the electron density map of the X-ray structure, may indicate that they are flexible. Superposition of AF2 prediction of Tli3^AIEC^ onto the crystal structure yields an r.m.s.d. of 1.4 Å (205/211 residues) (Figure 4B). The Tle3^AIEC^-Tli3^AIEC^ complex was predicted by AlphaFold2 multimer [24] (Figure 4C). Although the complex is roughly predicted, the position of Tli3^AIEC^ relative to Tle3^AIEC^ is displaced by ~5 Å outside of the cavity in the predicted structure of the complex, leading to reduced predicted interactions.

### 2.8. Tle3^AIEC^ Interacts with VgrG^AIEC^

**Figure 5:**
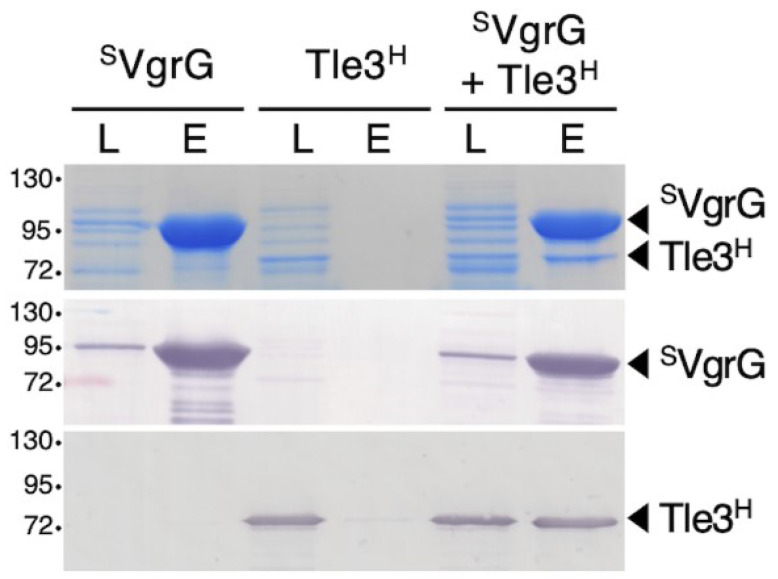
Tle3^AIEC^ interacts with VgrG^AIEC^. Strep-tag II-tagged VgrG (^S^VgrG) and 6His-tagged Tle3 (Tle3^H^) were produced separately from E. coli BL21 (DE3) pETDuet-^S^VgrG and BL21 (DE3) pRSF-Tle3^H^ cells, respectively. Lysates of cells (L) producing ^S^VgrG, Tle3^H^ or a 1:1 mix of ^S^VgrG and Tle3^H^ cell lysates (^S^VgrG + Tle3 ^H^) were incubated with Strep-Tactin Sepharose resin. After five washing steps, the Strep-tagged and co-precipitated proteins were eluted with desthiobiotin (E) and analyzed by SDS–PAGE and Coomassie blue staining (upper panel) or immunoblotting using anti-StrepII (middle panel) and anti-His (lower panel) antibodies. The molecular weight markers (in kDa) are indicated on the left and the positions of ^S^VgrG and Tle3^H^ are indicated on the right.

In EAEC, the Tle1 phospholipase was shown to be transported through its association to the VgrG1 spike protein. As for EAEC VgrG1 [18], the VgrG protein from AIEC (VgrG^AIEC^) carries a C-terminal extension (COG 4532 domain) comprising a DUF2345/TTR domain separated from the VgrG base by two helices. This domain was shown to be involved, together with the base of the VgrG needle β-prism, in the interaction of VgrG1 with Tle1 from EAEC [17,18]. The predicted structural organization of VgrG^AIEC^ and its genetic proximity to tle3 suggest a similar mode of binding. To test whether Tle3^AIEC^ associates with VgrG^AIEC^, we used a pull-down assay. Strep-tagII VgrG^AIEC^ (^S^VgrG) and Tle3^AIEC^ 6-His (Tle3^H^)-tagged proteins were produced separately and cell lysates were mixed and loaded on a Streptactin resin. SDS-PAGE followed by Coomassie blue staining and Western blot analysis of the eluted material showed that Tle3^H^ co-precipitated with ^S^VgrG (Figure 5), suggesting that Tle3^AIEC^ indeed interacts with VgrG^AIEC^.

### 2.9. Predicted Model of VgrG^AIEC^ and Docking with Tle3^AIEC^

**Figure 6:**
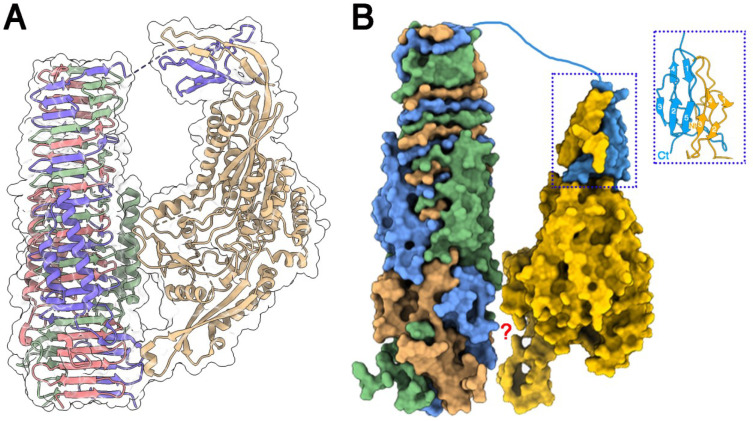
Structure of Tle1^EAEC^ and Tle3^AIEC^, and their interaction with VgrG^EAEC^ and VgrG^AIEC^. (**A**) Ribbon representation of the cryoEM structure of Tle1^EAEC^ in complex with VgrG^EAEC^. VgrG^EAEC^ is colored magenta, violet and green. Tle1^EAEC^ is colored beige. Surfaces are colored white and transparent. (**B**) Surface representation of the predicted structure of Tle3^AIEC^and VgrG^AIEC^, and a possible mode of interaction in their complex; inset: details of the interaction of VgrG^AIEC^ C-terminal adaptator with Tle3^AIEC^ N-terminal loop. VgrG^AIEC^ trimer is colored magenta, blue and green. Tle3^AIEC^ is colored yellow. The blue dot square identify the interaction between VgrG^EAEC^ TTR domain with the N-terminus loop of Tle3^AIEC^. The red question mark means that the interaction between the bVgrG^EAEC^and Tle3^AIEC^ cores remains unknown.

Comparison of the crystal structure of Tle3^AIEC^ (Figure 4A) with the cryoEM structure of Tle1^EAEC^ (Figure 6A) indicates that both structures share a α/β-hydrolase fold core of six β-strands surrounded by α-helices. Besides this core, the extra-parts of the structures diverge deeply. Two extensions that are absent in Tle3^AIEC^ crystal structure are observed in Tle1^EAEC^ at opposites sides (Figure 4A). However, these Tle1^EAEC^ extensions are present in the Tle3^AIEC^ AF2 prediction (Figure 4A) at similar positions; one of the two extensions also involves its N-terminus. Considering that the Tle1^EAEC^ extensions interact with VgrG^EAEC^ [18] (Figure 6A) and that we have shown that Tle3^AIEC^ interacts with VgrG^AIEC^, we hypothesized that a similar mode of interaction may occur for Tle3^AIEC^-VgrG^AIEC^. To explore this hypothesis, we co-predicted the VgrG^AIEC^ (residues 421-Ct) and Tle3^AIEC^ structures with AF2-multimer. As expected from the sequence similarity, both VgrGs exhibit close structures (Figure 6B). After a non-predicted linker (750–767), the predicted structure of VgrG^AIEC^ C-terminus domain, the adaptor (or TTR domain, 768–824), forms a complex with the N-terminus loop of Tle3^AIEC^ (Figure 6B-inset). It completes the two β-sheets of the Tle3^AIEC^ N-terminal loop by providing two β-strands to each of them. However, AF2 was not able to identify the second anchoring point of the complex, and the position of Tle3^AIEC^ was adjusted manually to resemble that of Tle1^EAEC^ in the complex with its VgrG (Figure 6B).

## 3. Discussions

In this study, we provide the first biochemical and structural characterization of a type VI phospholipase effector from the Tle3 family and its immunity protein Tli3. Tle3 effectors were identified by Russell et al. by bioinformatics [12]. *Pseudomonas aeruginosa* Tle3^PA^ was previously shown to be a periplasmic antibacterial T6SS effector, but its biochemical activity was not determined [35,36]. Here we show that Tle3^AIEC^ demonstrates PLA1 activity, inhibited by Tli3^AIEC^.

Tle3^AIEC^ was soluble and could be readily purified from the cytoplasm of *E. coli*. The absence of toxicity of Tle3^AIEC^ when produced in the cytoplasm of a host cell is in agreement with its predicted periplasmic activity. Indeed, Tli3^AIEC^ co-fractionates with the membrane fraction as a predicted outer membrane lipoprotein, suggesting a periplasmic activity for Tle3^AIEC^ that is common for Tle effectors.

The structure of the Tle3^AIEC^/Tli3^AIEC^ complex presented here is only the second structure of a Tle effector in complex with its immunity protein. The structure of Tle3^AIEC^/Tli3^AIEC^ reveals the inhibition mechanism, as a loop of Tli3^AIEC^ inserts deeply in the Tle3^AIEC^ substrate-binding crevice. This mechanism is very different from the “crab-claw like mechanism” used by Tli4 immunity protein to maintain Tle4 from *P. aeruginosa* in a closed conformation [37].

In EAEC, three Tle1s bind to the VgrG trimeric protein that serves as cargo for their delivery into the target cell. Each Tle1 interacts with the C-terminal transthyretin (TTR) extension of each VgrG monomer through its N-terminal domain extension, as well as on two additional zones of contact on the side and at the base of the VgrG needle. As expected from their sequence similarity, an AlphaFold2 model of VgrG^AIEC^ predicts a structure similar to VgrG^EAEC^, including two α-helices located alongside of the extended needle and a TTR domain, features corresponding to COG4532 domains found in several VgrG proteins [38]. Here we found that Tle1^EAEC^ and Tle3^AIEC^ share the small overall α/β mixed hydrolase fold core, and two divergent extensions, as modelled by AlphaFold2 for Tle3^AIEC^. The docking simulations of Tle3^AIEC^ on VgrG^AIEC^ suggest a similar mode of interaction, with an N-terminal domain/TTR adaptor interaction, and likely other contacts with the side of the needle as well as a contact with the base of the needle through the C-terminal extension of Tle3^AIEC^. We propose that effectors could have evolved different extensions to allow interaction with VgrG proteins, especially with the COG4532 domain-containing VgrG protein family.

## 4. Materials and Methods

### 4.1. Plasmid Constructions

*E. coli* AIEC LF82 chromosomal DNA was used as a template for all PCRs. *E. coli* strain DH5α was used for cloning procedures. Polymerase chain reactions (PCR) were performed using a Biometra thermocycler using Q5 High Fidelity DNA Polymerase (New England Biolabs, Herts, UK) or PfuTurbo (Agilent, Santa Clara, CA, USA). The genes encoding Tle3^AIEC^ and Tli3^AIEC^ (without the N-terminal 24-residue signal peptide) were amplified from AIEC LF82 genomic DNA using the primers listed in Supplementary Table S1. Tle3^AIEC^ was cloned, using the gateway technology, into the pETG20A vector that incorporates a Trx fusion protein followed by N-terminal His tag and a TEV cleavage site. The *tli3*^AIEC^ gene sequence corresponding to the mature form of Tli3^AIEC^ was cloned into the pET22b vector (Novagen, Madison, Wis, USA), fused to the PelB signal sequence, with a C-terminal 6-His tag in order to be addressed to the periplasm. Plasmids pET-Duet-SVgrG^AIEC^ and pRSF-Duet1-Tle3^H^ used for the pull-down experiments were constructed by a classical restriction/ligation method using the primers and restriction sites described in Supplementary Table S1. Plasmid pBAD33-Tli3^VSVG^ used for the fractionation experiment was constructed by restriction-free cloning, as previously described [39], using oligonucleotides introducing extensions annealing to the target vector (see Supplementary Table S1). The constructs were verified by sequencing (Eurofins, Nantes, France).

### 4.2. Expression, Production and Purification of Tle3^AIEC^, Tli3^AIEC^ and Tle3^AIEC^-Tli3^AIEC^ Complex

For Tle3^AIEC^ production, the expression plasmid pETG20A harboring the Tle3^AIEC^ coding sequence was transformed into *E. coli* T7 Iq pLys strain. Recombinant cells were grown in a 2 L Erlenmeyer flask at 37 °C in Terrific Broth medium supplemented with 100 µg/mL ampicillin. When the OD_600nm_ reached 0.8, the temperature was reduced to 17 °C and expression was induced overnight by the addition of 0.5 mM IPTG. Expression of selenomethionine (SeMet)-labelled Tle3^AIEC^ (Tle3^AIEC^ SeMet) was performed in *E. coli* T7 Iq pLys cells using the method of methionine biosynthesis pathway inhibition [40].

For Tli3^AIEC^ production, *E. coli* C41 (DE3) cells were transformed with pET22b-Tli3^AIEC^ for production in the periplasmic compartment and grown at 37 °C in Terrific Broth medium containing 0.1% glucose and 100 µg/mL ampicillin to an OD_600nm_ of approximately 0.8. Gene expression was then induced by the addition of 1 mM IPTG and the bacterial culture was further incubated for 16 h at 28 °C.

After induction, cells were harvested by centrifugation and stored at −80 °C. The Tle3^AIEC^ or Tle3^AIEC^ SeMet cell pellets were resuspended in Tris-HCl 20 mM pH 8.0, NaCl 300 mM, glycerol 5% (v/v), lysozyme (0.25 mg/mL), DNase (2 µg/mL) and MgSO_4_ 20 mM, and cells were lysed by ultrasonication on ice. The insoluble material was discarded by centrifugation at 20,000× *g* for 60 min at 4 °C. The soluble thioredoxin 6×His-tagged Tle3^AIEC^ or Tle3^AIEC^ SeMet fusion proteins were purified by affinity chromatography on a nickel–nitrilotriacetic acid resin (Bio-Rad) and the tag was removed after dialysis by overnight hydrolysis with the TEV protease and re-purified in presence of 10 mM imidazole. Pure fractions were concentrated by ultrafiltration using Amicon-Ultra 10-kDa cut-off and further purified by gel filtration chromatography (Superdex 200, 16/60 GE Healthcare) equilibrated in Hepes 10 mM pH 6.8, NaCl 150 mM using an AKTA purifier System (Amersham, Bath, UK).

Purification of Tli3^AIEC^ was performed from the periplasmic fraction prepared by osmotic shock, according to Skerra and Plückthun [41]. Briefly, the cell pellet was resuspended in 9 mL cold TES buffer (0.2 M Tris-HCl pH 8.0, 0.5 mM EDTA, 0.5 M sucrose) and kept on ice for 1 h. The periplasmic proteins were obtained by osmotic shock by addition of 13.5 mL cold TES diluted 1/4 into H_2_O, incubation for 1–2 h on ice, and w consecutive centrifugations at 20,000× *g* for 30 min at 4 °C. The Tli3^AIEC^ protein was then purified from the periplasmic fraction using the same procedure described above for Tle3^AIEC^. Production and purification of recombinant Tle1^EAEC^ was performed as previously described [17,18].

For the purification of the Tle3^AIEC^-Tli3^AIEC^ complex, the purified Tle3^AIEC^ and Tli3^AIEC^ were mixed with a molar ratio of 1:1.3. The complex was purified by gel filtration chromatography (Superdex 200, 16/60 GE Healthcare) equilibrated in a Hepes 10 mM pH 6.8, NaCl 150 mM buffer using an AKTA purifier System (Amersham, Bath, UK).

### 4.3. Phospholipase Activity Measurements. Enzymatic Assay on Synthetic Phospholipids

The activity of purified Tle3^AIEC^ was tested on fluorogenic phospholipid substrates (Figure 1A). Phospholipase A1 and A2 activities were monitored continuously using BODIPY^®^ dye-labeled phospholipids: PED-A1 (N-((6-(2,4-DNP)Amino)Hexanoyl)-1-(BODIPY^®^ FL C5)-2Hexyl-sn-Glycero-3-Phosphoethanolamine) 3 and red/green BODIPY^®^ PC-A2 (1-O-(6BODIPY-^®^558/568-Aminohexyl)-2-BODIPY^®^FLC5-sn-Glycero-3-Phosphocholine), respectively [42,43]. The sn-2 fatty acyl group in PED-A1 is a non-hydrolyzable alkyl chain, and PED-A1 substrate was used to specifically measure the PLA1 activity. The red/green BODIPY^®^ PC-A2 possesses an sn-1 uncleavable alkyl chain. Substrate stock solutions (50 µM) were prepared in ethanol. All enzyme activities were assayed in 10 mM Tris-HCl pH 8.0, 150 mM NaCl, 1 mM CaCl_2_ and 0.1% Triton X-100. Enzymatic reactions were performed at 20 °C for 25 min in a final volume of 200 µL containing 20 µg of Tle3^AIEC^ purified protein (from a 0.5 mg/mL stock solution) and 5 µM of the substrate. The release of BODIPY^®^ (BFCL5) (Life Technologies) was recorded at λexc = 485 nm and λem = 538 nm using a 96-well plate fluorometer (Fluoroskan ascent, Thermo Fischer Scientific, Waltham, MA, USA). Enzymatic activities were quantified using a BFCL5 calibration curve (0.08–200 pmoles in activity buffers) and expressed in pmol of fatty acid (or BFLC5) released per minute per mg of protein (pmol.min^−1^.mg^−1^). PLA1 from *Thermomyces lanuginosus* and bee-venom PLA2 (Sigma-Aldrich, Saint-Quentin Fallavier, France) were used as positive standards for PLA1 and PLA2 activities, respectively. Inhibition studies with Tli1^AIEC^ were performed by incubating 20 µg of Tle1^AIEC^ or Tle3^EAEC^ with various molar ratio of Tli1^AIEC^ (x_i_ = 0, 0.25, 0.5, 1 and 2). The residual activity was measured as described above.

### 4.4. Cytotoxic Effects of Tle1 and Tle3 Phospholipases on Mouse Macrophage

The cytotoxicity of the Tle1^EAEC^ and Tle3^AIEC^ phospholipases against eukaryotic cells was measured based on the reduction of resazurin as reporter of cellular viability [44,45,46]. Murine macrophage cells RAW264.7 (ATCC^®^ TIB-71™) were cultured in Dulbecco’s Modified Eagle’s medium (DMEM; Gibco, Thermo Fischer Scientific, Waltham, MA, USA). containing 4.5 g/L glucose, L-glutamine and sodium pyruvate supplemented with 10% heat-inactivated fetal bovine serum (Lonza, BioWhittaker^®^ sera, Walkersville, MD, USA), in the presence of 5% CO_2_, at 37 °C. For viability assays, cells were seeded in 96-well plates to a density of 1 × 10^5^ cells/well and, after 16 h, tightly attached macrophages were washed 1 time with 200 µL of complete culture medium. Then, fresh complete medium alone or fresh medium containing the purified Tle1^EAEC^ and Tle3^AIEC^ recombinant proteins (30 µg) was added into each well. After 16 h incubation, 20 µL of a 0.025% (*w/v*) resazurin solution was added to each well and fluorescence was measured following 4 h incubation at 37 °C and 5% CO_2_ in the dark, by excitation at 530 nm and emission at 590 nm using a TECAN Spark 10 M. Similar control experiments were carried out by treating the cells with the vehicle buffer, the pre-incubated Tle1^EAEC^-Tli1^EAEC^ inactive complex at stochiometric 1:1 ratio, as previously described [17], or with Triton X-100, used as lysis control.

### 4.5. VgrG^AIEC^-Tle3^AIEC^ Pull-Down Experiments

An amount of 100 milliliters of *E. coli* BL21 (DE3) culture cells transformed with pETDuet-^S^VgrG or pRSF-Tle3^H^ were grown to an OD_600_ of 0.8, and the expression of ^S^*vgrG* or *tle3*^H^ was induced with 0.5 mM of IPTG for 16 h at 16 °C. Cultures were centrifuged and resuspended in 1.5 mL buffer A (50 mM Tris-HCl pH8.5, 150 mM NaCl, 1 mM TCEP, cOmpleteTM™ protease inhibitors (Merck, Darmstadt, Germany ). Cells were lysed by sonication followed by a 30 min centrifugation at 20,000× *g* at 4 °C. Cleared lysates were then mixed (500 μL ^S^VgrG+ 500 μL Tle3^H^) or not (^S^VgrG alone, Tle3^H^ alone) for 1 h and then incubated with 100 μL of Strep-Tactin^®^ Sepharose^®^ resin (IBA Technologies) equilibrated with buffer A and gently mixed on a wheel at 4 °C. The resin was washed 5 times with buffer A and the proteins were eluted with buffer B (50 mM Tris-HCl pH 8.5, 250 mM NaCl, 1 mM TCEP, 2.5 mM desthiobiotin). In total, 1 μL of lysates and 7.5 μL of eluted fractions were analyzed by 10%-acrylamide SDS-PAGE followed by InstantBlue^®^ Commassie Blue Protein Stain (Abcam, Cambridge, UK) staining or immunoblotted using anti Strep (Mouse anti Strep-Tag Classic antibody, clone Strep-tag II, Biorad, MCA2489) or anti 6 × His (6 × His, His-Tag Monoclonal Antibody, Proteintech, 66005-1-Ig) antibodies and secondary antibodies coupled to phosphatase alkaline, and developed in alkaline buffer in presence of 5-bromo-4-chloro-3-indolylphosphate and nitroblue tetrazolium.

### 4.6. Crystallization of the Tle3^AIEC^-Tli3^AIEC^ Complex

The purified Tle3^AIEC^-Tli3^AIEC^ complex was subjected to crystallization screening assays with Hampton Research and Qiagen kits using the sitting-drop vapor-diffusion method at 20 °C. After 44 days at 20 °C, Tle3^AIEC^-Tli3^AIEC^ crystals were obtained using a reservoir solution consisting of 20% *v/v* PEG 3350, Bis Tris propane of pH 6.5 and 0.2 M sodium acetate trihydrate. The Tle3^AIEC^-Tli3^AIEC^ crystals’ quality were optimized by varying the pH, the protein and precipitant concentrations and the protein/precipitant ratio. The best condition was obtained by mixing 300 µL of protein solution and 100 µL of mother liquor (0.1 M Bis Tris propane pH 7.5, 25% PEG 3350, 0.2 M sodium acetate trihydrate). The Tle3^AIEC^-Tli3^AIEC^ crystals were soaked with different heavy atoms: CsI (quick shock), TaBr (3 daysshock), NaBr (quick shock) and cryocold in Ethylenglycol 12% to 14%. The best condition for rescreen of SeMet Tle3^AIEC^ SeMet-Tli3^AIEC^ crystals was in 70 mM Bis-Tris Propane, 30 mM Tris, pH 7.6 17% PEG 3350, 130 mM NaAcetate and 9.4% PEG 6000.

### 4.7. Data Collection and Processing

The SeMet Tle3^AIEC^/Tli3^AIEC^ data were collected at Proxima 2 using a Pilatus 6M detector at a wavelength of 0.9791 Å. Before data collection, the crystals were soaked in reservoir solution supplemented with 12% (*v/v*) ethylene glycerol for a few seconds and then flash-cooled in liquid nitrogen. All data were processed using XDS [47]. The final data-collection and processing statistics are provided in Table 1. A total of 375,390 measured reflections were merged into 78,970 unique reflections. The merged dataset was 98.9% complete to 3.6 Å resolution. The relevant data collection statistics are provided in Table 1.

### 4.8. Structure Determination and Refinement

Models of Tle3^AIEC^ and Tli3^AIEC^ and their complex were predicted with AlphaFold2 [23,24] on GitHub notebook (https://colab.research.google.com/github/deepmind/alphafold/blob/main/notebooks/AphaFold.ipynb#scrollTo=XUo6foMQxwS2) Stretches of residues with pLDDT < 80 were removed from the model Supplementary Figure S5. Molecular replacement was performed with the “cleaned” AlphaFold2 model using Molrep [29]. This starting model and subsequent ones were refined with Phenix [30], alternated with manual rebuilding using Coot [31]. The final statistics are provided in Supplementary Table S1. Figures were prepared with Chimera X [48].

## Figures and Tables

**Table 1:** Data collection and refinement statistics. Values in parenthesis refer to the highest resolution bin.

**Data Collection**	
Beamline	SOLEIL Proxima2
PDB ID	8BOZ
Space group	P2_1_
Cell parameters, a, b, c (Å)	67.9, 449.1, 116.2 β = 93.2°
Wavelength (Å)	0.9791Å
Resolution (Å)	48.7–3.6 (3.69–3.6)
Rmerge (%)	31 (160)
Mean ((I)/sd(I))	4.5 (0.82)
Cc (1/2)	97.6 (38.3)
No. of observations	305,990 (22970)
No. of unique reflections	78,970 (5814)
Completeness (%)	98.9 (99.3)
Multiplicity	3.9 (3.9)
**Refinement**	
Resolution (Å)	48.74–3.6 (3.64–3.6)
No. of reflections	78,957 (2660)
Atoms: protein/ions	45213/8
Rwork/Rfree (%)	23.4 (34.0)/26.8(37.6)
R.m.s.d. bonds (Å)/angles (°)	0.003/0.59
B-factors Wilson/model (Å^2^)	106/98
Ramachandran: favored/allowed/outliers (%)	90.6/8.1/1.3

## Data Availability

The predicted coordinates of Tle3, Tli3 and Tle3/Tli3 structures will be deposited in the open data repository Zenodo. The X-ray structure has been deposited with the Protein Data Bank with identifier 8BOZ.

## Supplementary Materials

The following supporting information can be downloaded at: https://www.mdpi.com/article/10.3390/ijms24021740/s1, Figure S1: Multiple alignment of Tle3 family members; Figure S2. Tli3 is a predicted outer membrane lipoprotein; Figure S3. Purification of the Tle3^AIEC^/Tli3^AIEC^ complex; Figure S4: AlphaFold2 pLDDT and PEA plots; Figure S5: Schematic strategy of molecular replacement; Table S1: Data collection and refinement statistics.; Table S2: Plasmids and primers.
