# Supplementary material for: Activity and Crystal Structure of the Adherent-Invasive Escherichia coli Tle3/Tli3 T6SS Effector/Immunity Complex Determined Using an AlphaFold2 Predicted Model
Source: Int J Mol Sci. 2023 Jan 16;24(2):1740. doi: 10.3390/ijms24021740 (PMC9860674; doi:10.3390/ijms24021740)

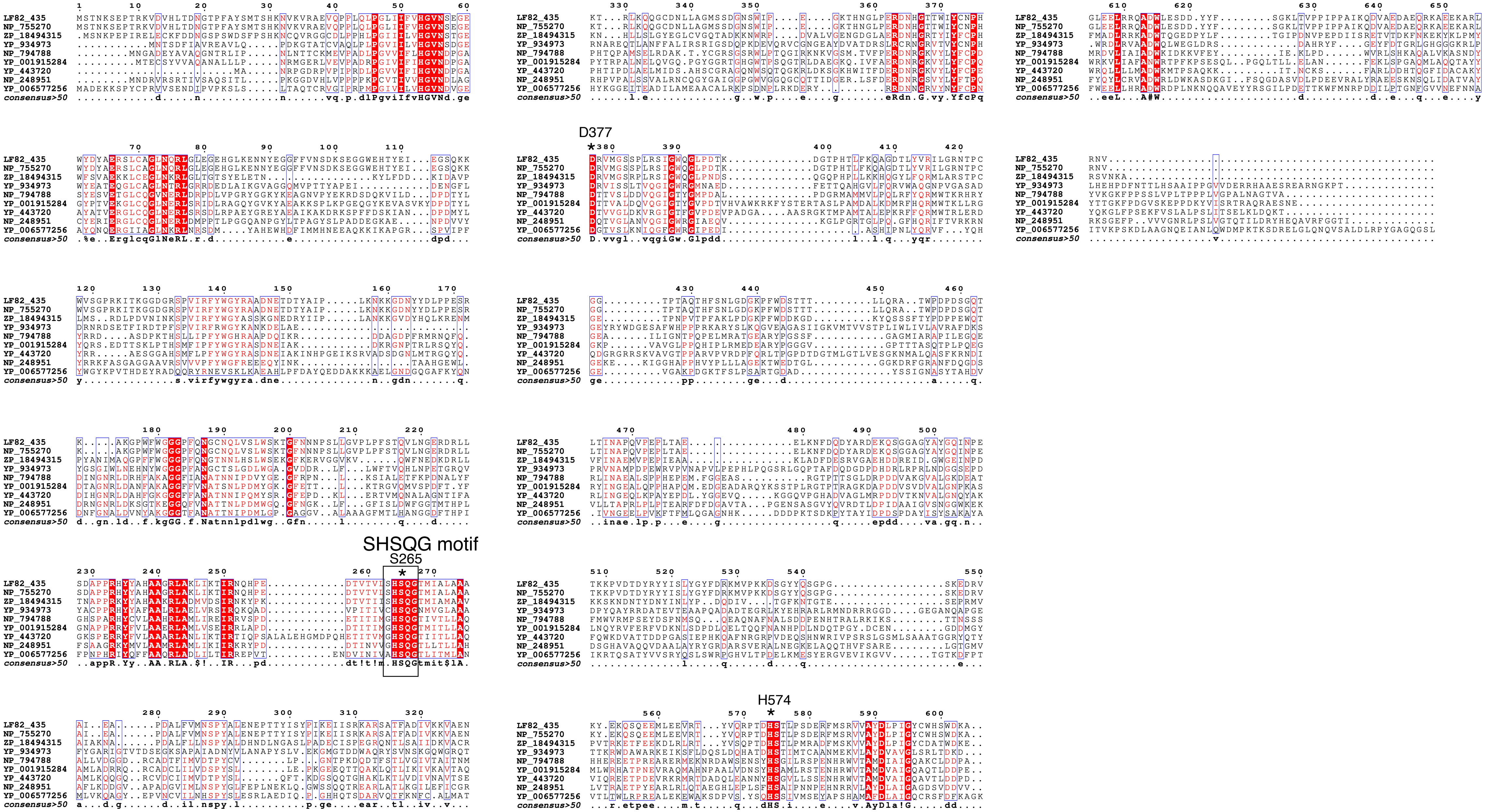

Figure S1. Multiple alignement of Tle3 family members. Sequence alignment of LF82_435 (Tle3[AIEC]) with representative members of Tle3 family identified in Russell *et al.*, 2013. The alignment highlights the GXSXG motif **(S/G/C/A)HSQG motif** of the Tle3 family, where the first G residue is replaced can be replaced here by S, C or A. The S265, D377, and H574 residues of the catalytic triad are indicated by stars. Figure prepared using MultAlin (Corpet *et al.*, 1988).

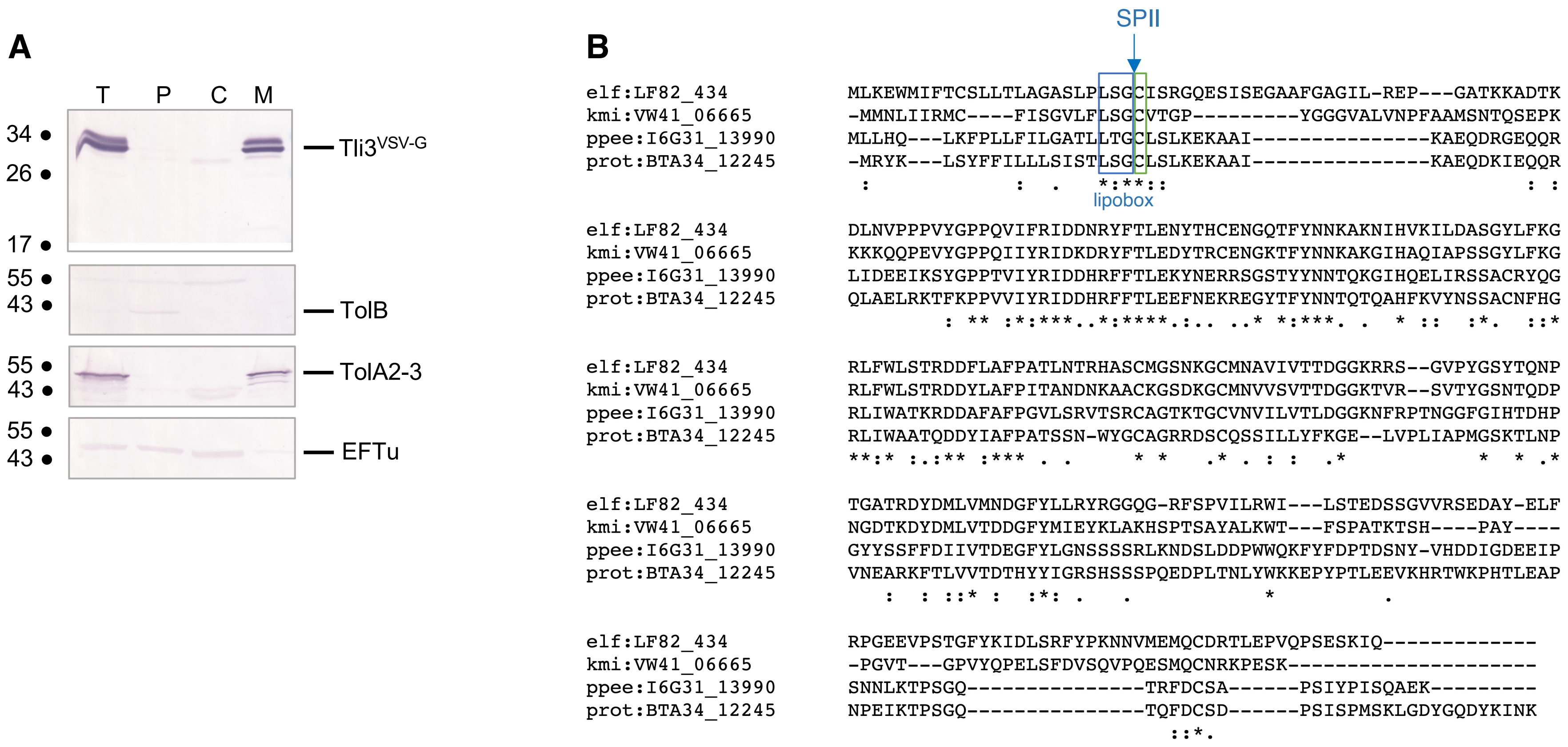

Figure S2. Tli3 is predicted outer membrane lipoprotein. A. DH5α cells producing VSV-G tagged Tli3 (T) were fractionated in periplasmic (P), cytoplasmic (C) and membrane (M) fractions as described previously (Aschtgen et al, 2008). Fractions were loaded on a 12.5% SDS-PAGE and immunoblotted using anti-VSV-G, anti-TolB (periplasmic control), anti-TolA-2-3 (membrane control) and anti-EFTu (cytoplasmic control) antibodies.

B. Sequence alignment of Tli3 orthologs using ClustalW. The putative lipobox predicted by SignalP 6.0 is indicated in blue. The arrow indicates the signal peptidase II predicted cleavage site. The N-terminal acylated Cys is indicated in green. (kmi:VW41_06665, Klebsiella michiganensis; ppee:I6G31_13990, Proteus penneri; prot:BTA34_12245, Proteus sp. CD3).

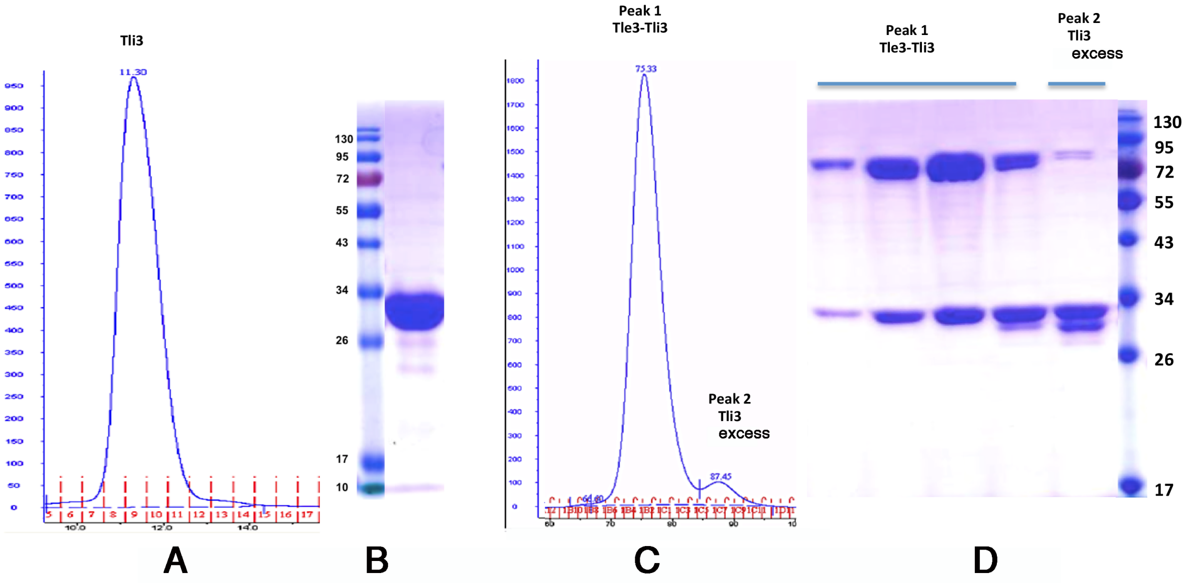

Figure S3: Gel filtration chromatography and SDS gels of the proteins under study. **A**: Gel filtration chromatogram of Tli3. **B**: SDS gel of Tli3 (right) with molecular weight markers on the left. **C**: Gel filtration chromatogram of the Tle3-Tli3 complex. **D**: SDS gels along the Tle3-Tli3 complex gel filtation fractions (left) and molecular weight markers on the right.

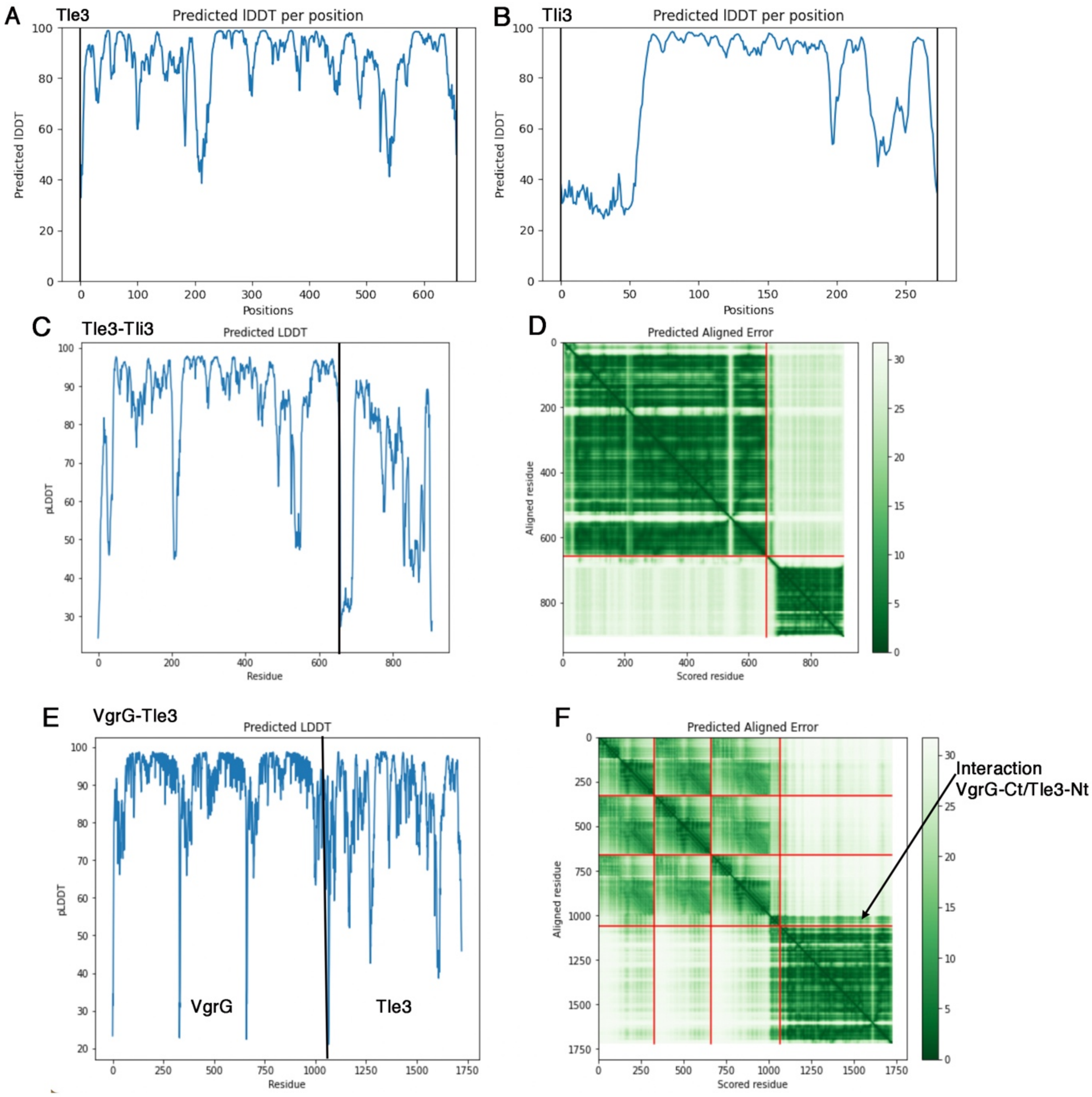

Figure S4. AlphaFold2 predicted local distance difference (pLDDT) and predicted aligned errors (PEA) output graphs. A: pLDDT of Tle3. **B**: pLDDT of Tli3. **C**: pLDDT of the Tle3-Tli3 complex (Tle3 left;Tli3 right). **D**: PEA of the Tle3-Tli3 complex. **E**: : pLDDT of the Tle3-VgrG$_3$ complex. **F**: PEA of the Tle3- VgrG$_3$ complex

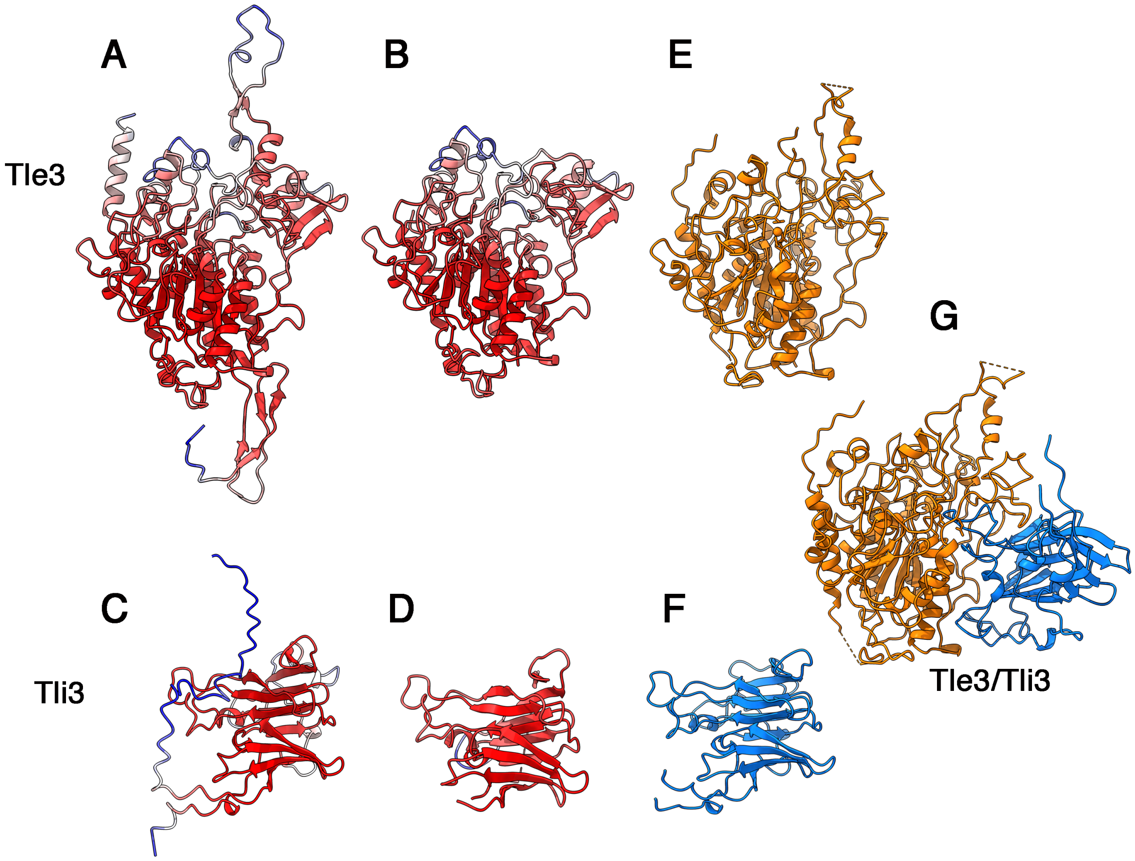

Figure S5. Molecular replacement strategy. A: The AlphaFold2 Tle3 prediction. **B**: The poorly predicted parts of Tle3 (white and blue in A) where removed yielding the Tle3 model used for molecular replacement. **C**: The AlphaFold2 Tli3 prediction. D: The poorly predicted parts of Tli3 (white and blue in C) where removed yielding the Tli3 model used for molecular replacement. **E, F, G**: The final refined structures of Tle3 (E), Tli3 (F) as present in the complex (G).

A B

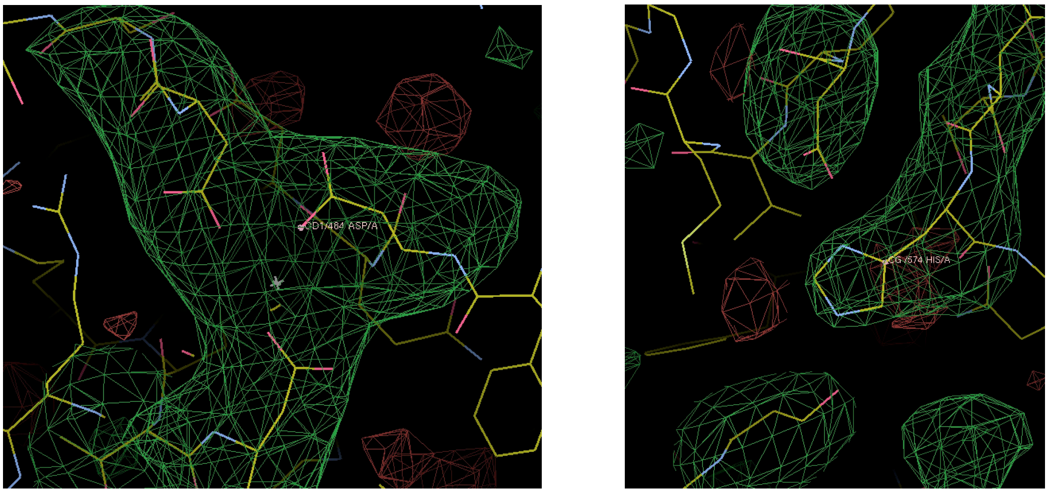

Figure S6. Omit maps around the Ca^{++} binding site and catalytic triad. The three catalytic amino-acids, the Ca^{++} ion and the three Ca^{++} binding Asp were deleted, and three cycles of full refinement with the remaining model were performed.

Supplementary Table 1. Plasmids and Primers

pETG20A-Tle3

5-attb1-LF82_435L

GGGGACAAGTTTGTACAAAAAAGCAGGCTTCATGAGTACCAATAAATCAGAGCCAA

C

3-attb2-LF82_435L

GGGGACCACTTTGTACAAGAAAGCTGGGTCTTACACATTACGTAACCGGGCC

pET-Duet-^{S}VgrG^{AIEC}

5-BamHI-^{STREP}VgrG

GATC**GGATCC**AAGGAGATATACATATG*TGGAGCCACCCGCAGTTCGAAAAA*GAGGCTCTTATGA

ACGTACAGTTCTTCGAC

3-HindIII-VgrG

GTAT**AAGCTT**TTATTTTTCAGAATAATTTTAGGTCATCAGATGCTTTTGACG

pRSF-Duet1-Tle3^{H}

5-NdeI-Tle3

GTCG**CATATG**AGTACCAATAAATCAGAGCCAACCCGAAAG

3-FseI-Tle3^{H}

GATC**GGCCGGCC**TTA*GTGGTGATGATGGTGATG*CACATTACGTAACCGGGCCTTTTCTTCTGC

pBAD33-Tli3^{VSVG}

5-pBAD- LF82 p434 -VSVG

ctctctactgtttctccatacccgtttttttgggctagcaggaggtattacaccATGTTGAAAGAATGGATGATATTTACG

3-pBAD- LF82 p434 –VSVG

ggtcgactctagaggatccccgggtaccTTATTTTCCTAATCTATTCATTTCAATATCTGTATATTGAATTTTACT

CTCTGAAGGTTG

[a] restriction site in bold

[b] StrepII-tag or His-tag coding sequence italicized

[c] optimized RBS sequence underlined

[d] sequence annealing to the target vector in lowercase

Uncropped gels

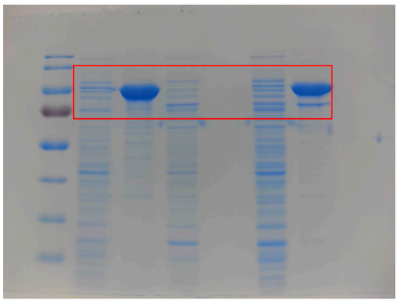

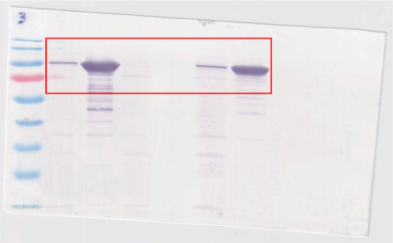

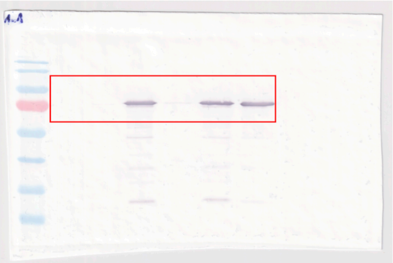

Supplement: Supplementary file 1 [file ijms-24-01740-s001.zip › ijms-2082730-supplementary.pdf]
